# Online Video-Mediated Compassion Training Program for Mental Health and Well-Being of University Students

**DOI:** 10.3390/healthcare12101033

**Published:** 2024-05-16

**Authors:** Thupten Tendhar, Melissa Ann Marcotte, Paul Bueno de Mesquita, Manob Jyoti Saikia

**Affiliations:** 1 Center for Nonviolence and Peace Studies, University of Rhode Island, Kingston, RI 02881, USA; thuptendar@uri.edu (T.T.);; 2 Department of Psychology, Rhode Island College, Providence, RI 02908, USA; 3 Department of Electrical, Computer and Biomedical Engineering, University of Rhode Island, Kingston, RI 02881, USA; 4 Department of Electrical Engineering, University of North Florida, Jacksonville, FL 32224, USA

**Keywords:** compassion, self-compassion, well-being, positive emotions, college students, video-mediated program

## Abstract

College students experiencing psychological distress have significantly greater negative emotions than students who practice compassionate thinking. We have developed Eight Steps to Great Compassion (ESGC), an innovative brief and no-cost online video training program about how to increase compassion among busy and young adult university students. To examine the effectiveness and benefits of the ESGC, a single-group pre-test–post-test quantitative design with undergraduate university students (*N* = 92; *M*_age_ = 20.39) evaluated its effects. The results from the post-test showed that the ESGC had a significant positive impact on increased feelings of compassion towards oneself, compassion for others, and the sense of personal well-being from the pre-test. The analysis of the PERMA-Profiler subscales also reflected a statistically significant increase in overall well-being and health and a decrease in negative emotions and loneliness. From the Post-Survey Lesson Feedback, 88% of the participants reported significant positive changes in themselves and the way that they live due to the program. These findings appear to show important implications for improving healthy minds and reducing negative emotions among university students.

## 1. Introduction

There is a growing need for broader educational and prevention approaches to address the root causes of mental health challenges facing students at post-secondary institutions of study, such as colleges and universities. Since the COVID-19 pandemic, students report increased feelings of anxiety, depression, and a lack of social support [1,2]. Unattended mental health problems can have a significant adverse impact on individual behavior, personal well-being, economic costs [3] and societal safety [4]. Specifically for students, poor mental health also negatively impacts their academic achievement and retention [5,6]. Many campuses for post-secondary study have adopted technology-enabled screening for mental health, but many still rely heavily on their limited counseling centers to address their students’ mental health [7]. Thus, responding to the mental health needs of students through widespread and accessible approaches is a priority for many higher education institutions.

Compassion education is one of the approaches that can address the root causes of personal and societal issues that impact people’s psychological and physiological well-being [8,9,10]. As evidenced by neuroscientists who study neuroplasticity, mental training such as mindfulness and compassion may result in transformative changes in the brain activity of individuals experiencing depression [11,12]. A review of compassion-based educational intervention programs and their effectiveness found a moderate effect size for improved satisfaction of life and reduced suffering due to anxiety, stress, and depression [13]. Further, developing psychological strengths, such as optimism and hope, increases the positive mental health of college students [14]. However, college students often have multiple obligations between school, work, and extracurricular activities, so being able to offer this mindfulness and compassion training digitally on demand is important. Although the use of digital wellness applications is increasing, college students report feeling overwhelmed by the ever-increasing number of apps that they already use and believe that they can destress by zoning out using entertainment apps and games [15], essentially the opposite of mindfulness. The current study explored the impact of a brief, video-based educational series on compassion created to help university students to learn about the topic and improve their overall well-being at their own time and convenience.

### 1.1. Compassion Education

The concept of compassion can be traced throughout the history of human evolution. It is rooted in spiritual, philosophical, theological, psychological, sociological, and biological perspectives [16,17]. From a Buddhist perspective, compassion is a regarded as sensitivity to the suffering of oneself and others, with a deep commitment to freeing beings from suffering and its causes [18,19]. Over the last two decades, this perspective has had a significant influence on the recent empirical research studies on compassion. According to one of the leading researchers on compassion, “it is necessary to open to their pain with mindfulness, respond with loving-kindness, and recognize interconnectedness in the experience of suffering in order to have compassion for another’s suffering, from a Buddhist perspective” [20]. Over the last two decades, this perspective has resulted in a notable increase in empirical research studies on compassion. The present study examined compassion and self-compassion based on a combination of contemporary Buddhism and Buddhist psychology.

Education can be thought of as an active process of enriching the human mind and heart that transforms perceptions, attitudes, and behaviors into constructive and purposeful forces toward the community’s betterment [21]. Many therapeutic, educational innovations in mind–body approaches and cognitive psychology include a new focus on topics including compassion [22,23]. Compassion education teaches us about the oneness of all sentient beings, including humans and animals. It emphasizes that we all have a shared desire to be free from suffering and enjoy happiness [24]. Therefore, compassion education offers a better understanding of how people are part of the world, a complex but vital relationship with animals and nature.

Compassion training was found to be a powerful method to improve both intrapersonal and interpersonal well-being [25]. Stanford University’s Center for Compassion and Altruism Research and Education found that domains of compassion, such as compassion toward self and compassion toward others, can be enhanced through a systematic training program for adults [26]. Even short-term compassion training significantly impacted prosocial behavior [27], which includes a broad range of actions such as comforting, helping, cooperating, and sharing to benefit one or more persons [28]. The increase in prosocial behavior can improve the well-being of those receiving compassion [29,30,31], as well as the person who is being compassionate. For example, Arimitsu and Hofmann [32] found that students who practice more compassionate thinking reported a lower frequency of negative emotions. A meta-analysis of 21 randomized controlled trials consisting of 1285 individuals who participated in interventions designed to cultivate compassion toward self and others revealed a significant positive change in scores with moderate effect sizes [33]. These positive changes have included reduced feelings of stress and anxiety [34], increased positive moods [27,35], and even neural changes associated with love and positive valuation [29].

Interventions that reduce stress and increase self-compassion may positively impact college students’ behavioral and psychological well-being [36]. Despite these benefits, compassion education and training curricula remain largely inaccessible for many college students due to the program’s length, costs, teaching schedules, and methods. Typical existing compassion programs are designed for in-person group instruction, ranging from 8 to 12 weeks in length, requiring hours of participation and completing homework and practice assignments. Many students are reluctant to engage with such programs, given the demands of their required coursework and financial needs for employment. Therefore, there is a clear need for more individualized, flexible, streamlined, time-efficient, and convenient compassion training programs. These practical barriers have led to the exploration of online alternatives.

Internet-based interventions and education programs promoting positive psychological states of mind are receiving greater attention. For example, an experimental study promoting happiness with 435 adults using video-based online instructions reported a significant increase in participants’ subjective well-being compared to the control group [37]. Thus, online internet methods offer expanded possibilities for enhancing self-compassion and compassion for others. However, few online programs have focused on compassion for both self and others [38].

### 1.2. Present Study

The purpose of the present study was to address the need for short-term, no-cost online compassion training programs specially designed for busy young adult university students. This study examined the effects of an innovative brief video-mediated education program to increase self-compassion, compassion for others, and a sense of personal well-being. Completing the Eight Steps to Great Compassion [21] program was expected to improve self-compassion, compassion for others, and personal well-being. Moreover, we expected that participants in this study might find this compassion program practical and helpful when delivered as a short-term online individualized program.

## 2. Methodology

A sample of undergraduate university students volunteered to participate in this compassion education online training from a public university in the northeastern region of the United States. All participants were pre-tested to understand their self-described knowledge and experience with compassion. A post-test was used to measure any increase in participants’ sense of well-being and compassion towards themselves and others. There were 101 volunteering students who completed the pre-survey, eight lessons, and the post-survey. Because of the age criteria and missed deadlines, the final sample included 92 individuals ages 18–24 years old. Students had various areas of study and were representative of the ethnic and cultural diversity of this university (Table 1). According to the university’s Intuitional Review Board (IRB) requirements, all research procedures including informed consent and anonymous participation were observed.

### 2.1. Study Design

A single group pre-test–post-test design was employed [39]. The independent variable was an eight-lesson, video-mediated compassion education training program titled Eight Steps to Great Compassion designed for this study. The three dependent variables were measured via the self-compassion, compassion for others, and well-being surveys.

### 2.2. Eight Steps to Great Compassion

The Eight Steps to Great Compassion program [21] was developed by a Buddhist scholar with support from the University of Rhode Island’s Center for Nonviolence and Peace Studies. The program is grounded in a Buddhist perspective and incorporates a social learning, video-mediated, online methodology. The foundation of the entire curriculum was based on Buddha Maitreya’s Seven-Point Cause and Effect Instruction. The instruction was taught in classical Buddhist texts such as A Lamp for the Path of Awakening by the eleventh-century Indian scholar Atisha Dipamkara Shrijñana [40] and Lam Rim Chenmo by the fourteenth-century Tibetan scholar Tsong-Kha-pa Lobsang Drakpa [41]. The curriculum was organized into eight lessons that were derived from theory, research, and practice. Lesson topics in order of presentation were (1) mindfulness, (2) Common Humanity, (3) Gratitude, (4) Loving-Kindness, (5) Empathetic Concern, (6) Forgiveness, (7) self-compassion, and (8) compassion for others. These lessons were scaffolded and presented logically to support a more in-depth understanding of compassion. Each video lesson consisted of approximately 6–10 min of interactive instructional information on one of the eight topics. The structure of each lesson was the same: (1) Introduction, (2) Concept Explanation, (3) Narrated Video and Photographic Depiction, (4) Practice Exercise, and (5) Inspirational Quote. The beginning of each lesson also included a connection to the preceding lesson.

The methodology is grounded in the Social Learning Theory and Observational Learning principles proposed by Albert Bandura in the early 1960s. In a series of research studies, Bandura and colleagues demonstrated that when children and others learned aggressive behavior patterns through video-mediated imitation and modeling processes, they exhibited specific aggressive behavior in subsequent situations [42]. Since then, many studies have used Social Learning Theory as a framework for successfully learning prosocial behavior [43].

### 2.3. Procedures

The Eight Steps to Great Compassion online program began with an introduction, followed by eight individual lessons: mindfulness, Common Humanity, Gratitude, Loving-Kindness, Empathetic Concern, Forgiveness, self-compassion, and compassion for others [21]. Participants could sign into their online university account from anywhere to access and view their compassion video lessons at any time of the day. Each video lesson was between five and 10 min in length. All lessons were sequential. Watching Lesson One was a prerequisite to proceeding to Lesson Two and so forth. Participants were encouraged to watch only one lesson a day but were allowed one month to complete all the lessons at their own pace. Each lesson concluded with a practice assignment to apply what was learned. A reminder email with an encouraging photographic message was sent once a week to participants who appeared to be progressing slowly with their lessons. Only after watching all the lessons were students able to complete the post-survey. The post-survey asked about compassion and well-being and included questions about the Eight Steps to Great Compassion program’s length, relatability, helpfulness, and effectiveness [44]. A thank-you email with a completion certificate was sent to students who completed all eight lessons and both surveys.

## 3. Measures

### 3.1. Self-Compassion Scale (SCS)

The SCS was used to assess compassion towards the self. The SCS [45] consists of 26 items that assess the positive and negative aspects of three main self-compassion components and contribute to an overall self-compassion score. The SCS consists of the following six subscales: Self-Kindness, Self-Judgment, Common Humanity, Isolation, mindfulness, and Over-Identification. This instrument has been used in previous research and is an established measure of self-compassion [46,47]. The SCS has high internal consistency between items and the total score, with A Cronbach alpha of α = 0.87 [48]. The high consistency of the SCS total score has also been reported by Van Dam et al. [49], α = 0.92; Neff et al. [50], α = 0.90; and Raes [51], α = 0.90. The SCS has good test–retest reliability over time, with test–retest reliability coefficients of r = 0.93 for three weeks [45] and r = 0.71 for five months [52].

### 3.2. Compassion towards Others Scale (CS)

The CS was utilized to assess compassion towards others. The CS consists of 24 items with a five-point Likert scale (1 = almost never to 5 = almost always) response format and is based on the same foundational theory of compassion as the SCS [53]. The CS measures the following factors: Kindness vs. Indifference, Common Humanity vs. Separation, and mindfulness vs. Disengagement. Previous studies support the validity and reliability of this instrument [54,55]. Good reliability of the CS is also indicated by a Cronbach alpha of 0.90 and a split-half coefficient of 0.90 [53].

### 3.3. PERMA-Profiler Scale

The PERMA-Profiler Scale was utilized to measure well-being. This instrument is based on Seligman’s positive psychology theory of well-being and is a recognized measure of flourishing among adults [56]. Many psychologists and social scientists use the term “flourishing” to describe high levels of well-being [57]. According to Seligman, there are five main well-being elements: positive emotion (P), engagement (E), relationships (R), meaning (M), and accomplishment (A) [58]. The PERMA-Profiler Scale contains 23 items related to these five well-being elements that are categorized into four subcategories: well-being, negative emotion, health, and loneliness. The PERMA-Profiler questions are rated on an 11-point scale ranging from 0 to 10. The negative emotion and loneliness subscales are reverse-scored to show that higher scores indicate better mood and social connection. The PERMA-Profiler Scale has been shown to successfully measure the four PERMA domains with acceptable internal reliability (α = 0.53–0.95), test–retest reliability (α = 0.51–0.88), and support for divergent and convergent validity [56].

### 3.4. Post-Survey Program Feedback

Participants were asked to respond to 13 feedback items as the final part of the post-survey to assess the face validity of the lessons. These items asked participants to rate how realistic, relatable, helpful, effective, and beneficial the lessons were for them. Participants were also asked how and when they viewed or revisited any lessons, how much or how little they practiced each lesson, and if this program led to positive changes in themselves and how they live overall (Table 2).

## 4. Results

### 4.1. Pre–Post Differences for Total Self-Compassion

A paired-samples *t*-test examined the change in the mean total score on the Self-Compassion Scale between the pre- and post-surveys. There was an increase from the pre-test (M = 74.40, SD = 16.94) to the post-test (M = 91.00, SD = 18.16), a statistically meaningful difference of 16.598 points, with a 95% CI [13.577, 19.618], *t*(91) = −10.91, *p* < 0.001, and Cohen’s *d* = 1.138. This result indicates that university students who completed Eight Steps to Great Compassion had a large increase in their compassion towards themselves.

### 4.2. Pre–Post Differences for Self-Compassion Subscales

Paired-samples *t*-tests were performed to examine whether there were significant changes in each of the six subscales of the Self-Compassion Scale: Self-Kindness, Self-Judgment, Common Humanity, Isolation, mindfulness, and Over-Identification. The results are listed in Table 3 and show that university students who completed Eight Steps to Great Compassion significantly increased their scores across all six self-compassion subscales.

### 4.3. Pre–Post Differences for Total Compassion for Others

A paired-samples *t*-test examined the changes in mean total scores on the Compassion Scale between the pre- and post-surveys. There was an increase from the pre-test (M = 99.54, SD = 12.15) to the post-test (M = 104.54, SD = 11.64), a mean increase of 5 points, with a 95% CI [3.144, 6.856], *t*(91) = −5.35, *p* < 0.001, and Cohen’s *d* = 0.558. This result indicates that university students who completed Eight Steps to Great Compassion had a moderate increase in their compassion towards others.

### 4.4. Pre–Post Differences for Compassion for Other Subscales

Paired-samples *t*-tests were performed to examine whether there were significant changes in each of the six subscales of the Compassion Scale: Kindness, Indifference, Common Humanity, Separation, mindfulness, and Disengagement. The results are listed in Table 3 and show that university students who completed Eight Steps to Great Compassion significantly increased their scores across all six compassion for others subscales.

### 4.5. Pre–Post Differences for Total PERMA Well-Being

A paired-samples *t*-test examined the change in the mean total score on the PERMA-Profiler Scale between pre- and post-surveys. There was an increase from the pre-test (M = 147.32, SD = 35.00) to the post-test (M = 164.72, SD = 35.00), a 17.402 point increase, with a 95% CI [13.074, 21.73], *t*(91) = −7.98, *p* < 0.001, and Cohen’s *d* = 0.833. This result indicates that university students who completed Eight Steps to Great Compassion had a large increase in their sense of well-being.

### 4.6. Pre–Post Differences for Well-Being Subscales

Paired-samples *t*-tests were performed to examine whether there were changes in each of the four subscales of the PERMA-Profiler: (i.e., well-being, negative emotions, health, and loneliness), as well as in the overall total. The results indicated that there was a statistically meaningful (*p* < 0.001) improvement in all domains (see Table 4). Specifically, there was a medium–large effect on overall well-being, a medium effect on relieving negative emotion, and small–medium effects on improving health and relieving loneliness.

### 4.7. Post-Survey Program Feedback

**Figure 1:**
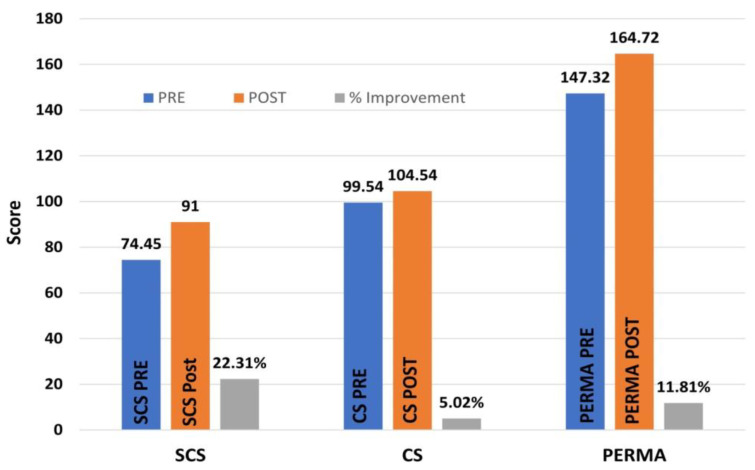
Percentage of improvement in total self-compassion (SCS), compassion for others (CS), and well-being (PERMA) after completing the online Eight Steps to Great Compassion lessons.

**Figure 2:**
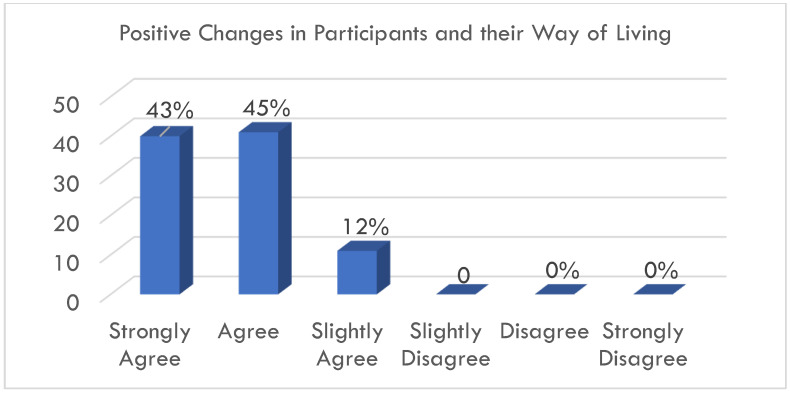
Percentage of participants who agreed that the video-mediated Eight Steps to Great Compassion lessons led to positive changes in themselves and the way in which they live.

Participants were asked to rate the effectiveness of each lesson in the Eight Steps to Great Compassion program on a five-point response scale (1 = very ineffective, 5 = very effective). The overall mean effectiveness rating was 4.44. When rating the impact of the compassion education training program on their personal change, 88% of participants either agreed or strongly agreed that completing the lessons led to positive changes in themselves and in the way that they live, as shown in Figure 1. The remaining 12% of participants also agreed, albeit slightly, with the positive impact of the lessons (Figure 2).

## 5. Discussion

The goal of this study was to evaluate the effects of an innovative online compassion education program. The primary research questions focused on whether or not there would be increases in compassion for the self, compassion toward others, and overall well-being due to participating in the online video-mediated compassion education program. Feedback from participants, including that about the effectiveness, helpfulness, and benefits of the training, was also collected for overall program assessment. Overall results demonstrated that the Eight Steps to Great Compassion online program positively impacted participants’ compassion towards themselves and others and their sense of personal well-being.

For the university students who completed this program, mean scores for total self-compassion significantly increased by 22.31%, and mean scores for total well-being significantly increased by 12%. These findings show that even eight educational lessons consisting of five-to-ten-minute-long instructional videos can improve participants’ compassion. These findings also are consistent with previous research that found that compassion can be enhanced through training [26], compassion for others can be developed [59], and compassion is an essential predictor of well-being [60].

This study also detected significant relationships between the primary outcome variables. First, change scores for total self-compassion and total well-being were positively correlated. This finding is consistent with previous work by Verma and Tiwari [61] that reported the significant role of self-compassion in one’s overall sense of well-being. Second, change scores for total self-compassion and total compassion for others were also positively correlated. This finding might corroborate the notion that it becomes easier to be compassionate towards others when one possesses self-compassion, especially when dealing with adverse events in life. In contrast, change scores for total compassion for others and total well-being did not appear to be related. This might indicate that one can still treat others with compassion even when one’s well-being is not in a positive state.

Compassion education training programs, such as Eight Steps to Great Compassion, that effectively increase compassion and well-being could benefit the lives of university students and those around them for additional reasons. On an individual level, enhanced self-compassion may also improve emotional intelligence and resilience, as others have found associations with these factors [62,63]. On an interpersonal level, enhanced compassion for others could help because it aspires not to harm [64]. By developing compassion, students create a more inclusive campus environment conducive to positive, cooperative learning and development.

### 5.1. Changes in Self-Compassion Components

Significant pre–post changes in self-compassion subscale scores indicated improvement in self-compassion by program participants. These changes can impact student success both academically and emotionally.

The significant increase in Self-Kindness is consistent with [65] report that university students with self-compassion treat themselves with Kindness rather than with self-criticism. In this way, a benefit of Self-Kindness is that it could help university students to prepare for their emotional and academic demands [54]. The significant change in mindfulness has particular importance for university students. As Keye and Pidgeon [66] have found, both mindfulness and academic self-efficacy substantially impact university students’ resilience and success by enhancing mindfulness. The Eight Steps to Great Compassion program may improve resilience among university students and reduce university dropouts.

The significant reduction in Self-Judgement could be protective against eating disorders. First, reducing Self-Judgment through the Eight Steps to Great Compassion program contributed to an overall increase in self-compassion, negatively associated with shaming and eating disorders [67]. Second, practicing self-compassion has been found to reduce eating disorders [68]. The significant increase in Common Humanity is aligned with previous studies that reported significant associations between self-compassion and shared humanity [69,70] and between higher self-compassion and better perspective-taking [46]. The significant decrease in Isolation is consistent with the negative correlation between self-compassion and Isolation and the positive correlation between self-compassion and a sense of community among adolescents reported by [69]. An example of an Isolation subscale item used in the current study is “When I fail at something that’s important to me, I tend to feel alone in my failure” [71]. By reducing feelings of Isolation, the program helps to create a stronger sense of belonging with others.

The significant decrease in Over-Identification is consistent with the work of Albertson et al. [72] that showed meditation on self-compassion decreased Over-Identification. An example of an Over-Identification subscale item used in the current study is “When I am feeling down, I tend to obsess and fixate on everything that’s wrong” [71]. Decreasing Over-Identification due to meditation on self-compassion [72] could help university students to prevent stress, anxiety, and selfish attitudes.

### 5.2. Changes in Components of Compassion for Others

Significant pre–post changes in compassion subscale scores indicated improvement in compassion towards others among program participants. The significant increase in Kindness could help to make university campuses safer places by reducing the harmful and violent behaviors of students towards others around them. The significant reduction in Indifference may give students the space to be more sensitive to the feelings, needs, and well-being of others. This ability to understand and share in others’ feelings is empathy, and it has been identified as one of the twelve elements of emotional intelligence [73]. As it reduces Indifference, the Eight Steps to Great Compassion program enables participants to be more empathetic human beings who can better appreciate the perspectives of those in both desirable and undesirable life circumstances.

The significant increase in Common Humanity reflects the success of this compassion education program’s efforts to create a sense of oneness with and belonging to the same human family. A student body unified in this way would be an invaluable asset in overcoming issues of inequality, injustice, and discrimination that divide university campuses. The significant decrease in Separation occurred with an increase in the feeling of Common Humanity that involves a sense of connectedness to other people. This is consistent with the negative relationship between Separation and connectedness found by Ingoglia et al. [74]. Feeling less separated and more connected would help to reduce loneliness, which has been linked to psychological stress, poor sleep, and adverse physical health [75,76]. Thus, colleges that use Eight Steps to Great Compassion could help to reduce feelings of separateness among students, which would be beneficial for those students’ physical and mental health.

The significant increases in mindfulness in both the Self-Compassion Scale and Compassion Scale suggest greater awareness about thoughts and feelings, words, and actions towards the self and others. Equipped with this heightened state of consciousness, students who complete the Eight Steps to Great Compassion program can act with more care to avoid using hurtful language and engaging in harmful behaviors, thus contributing to a more compassionate campus community.

The significant decrease in Disengagement, similar to the decrease in Separation, could benefit students by reducing loneliness. In addition, people who are less disengaged from life and their community are more likely to interact with others and create connections with them. This type of behavior would help students to improve interpersonal relationships and better understand themselves and those around them.

### 5.3. Changes in Well-Being

Significant pre–post changes in PERMA-Profiler subscale scores indicated improvements in well-being among program participants. Various components of well-being also showed significant changes. After viewing the lessons, participants had significantly higher feelings of happiness and could strongly impact students’ quality of life and academic gain. This finding was similar to previous studies that reported a positive association between social relationships and well-being [77] and compassion as a crucial component for human flourishing [78]. Happiness has been considered the ultimate motivation and the highest good for human actions by philosophers throughout history [79]. Furthermore, a happier, flourishing student would be less likely to engage in violent or harmful thoughts or behaviors. A significant change in overall well-being was related to previous findings that found compassion is an important predictor of a person’s sense of well-being (e.g., [60]). By improving students’ overall well-being, colleges and universities could help to create a positive and healthy learning environment for all, as envisioned by many education philosophers, with compassion education.

The significant change in negative emotions as a detracting component of well-being implies that this brief online video-mediated compassion education might help to reduce students’ negative emotions, which can harm students’ health and learning outcomes. This finding is significant because it supports emotions’ vital roles in learning, developing behaviors, interactions, and relationships in higher education [80]. Above all, emotions determine the quality of human lives, and they often direct how people think, speak, or act [81]. The finding also is consistent with the literature that found that increased compassion causes reductions in worry and emotional suppression [82].

The reported change score in health was also very significant at the post-survey time. University students completing the entire Eight Steps to Great Compassion program reported significant increases in their physical health. This remarkable finding is further supported by the research literature reporting that compassion significantly influences physical health [83]. Although it was not feasible in this study to collect data on or examine university students’ health, these results indicate that health improvements, as reported by these research participants, may result from brief, online, video-mediated compassion education interventions and prevention programs.

Finally, a significant decrease in reported feelings of loneliness that previous research has associated with anxiety, poor sleep, and depressed moods was noted. Loneliness also predicted more inadequate social interaction, higher aggressive behaviors, and more addictive substance use among college and university students in twenty-five countries [84]. Thus, loneliness is recognized as one of the causes of poor health among college students [85]. Researchers found that better physical and mental health correlated with lower loneliness among 20,096 U.S. participants who were 18 years old or older [86]. One implication of this finding is that colleges could help to uplift students’ moods, reduce anxiety, and increase health by providing opportunities to learn compassion through video-based formats such as the Eight Steps to Great Compassion Program, which is easily accessible and convenient and individualized online.

## 6. Conclusions

This was a descriptive exploratory study of the benefits of compassion education using a brief video-mediated delivery modality. The university students who completed this brief, online compassion education program experienced significantly increased self-compassion, compassion towards others, and well-being. At the same time, they reported fewer negative emotions, better health, and more happiness. These results are consistent with the Buddhist view of compassion, which defines compassion as sensitivity to the suffering of self and others, combined with a deep commitment to relieve it. The success of the video-mediated format of the training program, through which participants observed a compassion concept and practiced it, is consistent with Bandura’s early social learning theory of observational behavioral modeling [87,88]. In contrast to Bandura’s study on role modeling and film-mediated aggressive behaviors, this unique and innovative approach is a welcomed reversal and shift to a needed positive refocusing. It focused on developing constructive attributes and expanding and advancing approaches to compassion education leading to positive outcomes.

Dewey [89] proposed that education is the means to achieve the “social continuity of life”, and society’s existence depends on processes for the transmission of values as much as its biological life. Today, however, a significant number of young adult university students may be experiencing personal and mental health challenges due to a lack of knowledge and understanding about self-compassion or little experience of the regular practice of genuinely extending compassion toward others. The survival and success of this young adult generation are essential for the future of society. Therefore, it is crucial to find ways to embed compassion as the essence of all education and education policy for the healthy continuity of individuals and the formation of a more compassionate and flourishing society.

## 7. Strengths and Limitations

This study delivered a novel compassion education intervention and efficiently assessed its effectiveness using a quasi-experimental pre–post research design to quantitatively test the main dependent variables and results of the online video training within a short timeframe. Because the online video lessons were free, individualized, and conveniently accessible, study volunteers learned and practiced compassion at their own pace without interference with their coursework and jobs. Since participants had different genders, ethnicities, ages, college years, religious beliefs, and sociodemographic backgrounds, the sample was representative of a medium-sized public university.

Five main limitations of the present study should be recognized. First, although the quasi-experimental pre-test–post-test design is a popular frequently employed method, it raises questions regarding external validity and limited causality. Second, the study utilized a convenience sample without the possibility of random selection. Consequently, the findings may only apply to this particular group and may not be generalizable to university student populations more broadly. Third, only the immediate impact of the training was studied, and the long-term effects of this brief online program over time were not assessed. Fourth, there was no control group for comparison, so we cannot say with certainty that simply filling out the compassion scales increased the participants’ compassion. Finally, observations and interviews were not conducted; therefore, reliance only on self-report survey data is acknowledged as a potential source of social desirability bias [90].

## 8. Future Directions

Several areas of future research merit special attention for their potential to expand the use and enhance the benefit of our compassion education program. First, a Pre-Test–Post-Test Control Group Design and comparison among undergraduate and graduate students to examine the effects of compassion intervention would yield more generalizable results. Also, randomization for the selection and assignment of participants would address bias and threats to validity. Similarly, future research might include young children and adolescents, parents, and seniors representing members of broader populations and determine the influence of compassion training across different developmental stages. Relatedly, a longitudinal follow-up study design could help to ascertain if the improvements in compassion and well-being persist and, if so, for how long. One of the most promising possibilities for future research is the adaptation of the Eight Steps to Great Compassion from online learning management systems to social media applications compatible with different devices such as tablets and smartphones. Expanding compassion training into popularly used existing technologies would enable the study of its use by and its benefits for large numbers of people across the world who have access to these devices.

Finally, future studies also could consider questions related to the transfer of learning and behavior change. Questions regarding the transfer of learning and sustained behavioral change remain to be explored further and are fertile ground for future research. For example, to what extent does the training program result in the application of compassion to daily real-world situations? Furthermore, more research exploring a theoretical perspective of behavioral change underlying compassion education would be valuable in understanding appropriately matching training and instructional formats to different populations and cultures. For instance, behavior change education and intervention programs have been shown to be most successful when following a transtheoretical model of behavior change [91]. According to this model, changing a person’s level of compassion for the self and others first requires an assessment of the person’s readiness and current stage of change to match the most compatible instructional intervention that will move the person forward to the next stage of change and sustain changes in compassion in the long term.

## Figures and Tables

**Table 1:** Categorical demographics.

	n	%
**Sex**		
Female	79	86%
Male	12	13%
Non-Binary	1	1%
**Race/Ethnicity**		
Asian	7	7.60%
Black	5	5.40%
Biracial	11	12%
Hispanic	4	4.30%
White	65	70.70%
**College Year**		
Freshmen/First Year	19	20.70%
Sophomore/Second Year	11	12%
Junior/Third Year	26	28.30%
Senior/Fourth Year	26	28.30%
Fifth or Sixth Year	10	11%
**Religiosity**		
Religious	43	46.70%
Non-religious	49	53.30%

**Table 2:** Correlations between scale total scores pre- and post-test.

	Self-Compassion (Pre)	Compassion for Others (Pre)	PERMA (Pre)	Self-Compassion (Post)	Compassion for Others (Post)
Compassion for Others (Pre)	0.173	-			
PERMA (Pre)	0.735 **	0.210 *	-		
Self-Compassion (Post)	0.657 **	0.099	0.482 **	-	
Compassion for Others (Post)	0.212 *	0.717 **	0.272 **	0.300 **	-
PERMA (Post)	0.647 **	0.136	0.817 **	0.688 **	0.265 *

Note. * *p* < 0.05, ** *p* < 0.01.

**Table 3:** Self-compassion and compassion for others: total and subscale paired-sample results.

			95% CI of the Difference		
Self-Compassion (SC)	Mean Difference	*SE*	LL	UL	Cohen’s *d*
Self-Kindness	3.272 **	0.340	2.597	3.947	1.004
Self-Judgment	3.098 **	0.400	2.303	3.893	0.807
Common Humanity	2.000 **	0.372	1.261	2.739	0.560
Isolation	2.924 **	0.363	2.202	3.645	0.839
Mindfulness	2.674 **	0.290	2.098	3.250	0.961
Over-Identification	2.630 **	0.309	2.017	3.244	0.887
Total SC	16.598 **	1.521	13.577	19.618	1.138
Compassion for Others (CO)					
Kindness	0.728 **	0.207	0.316	1.140	0.366
Indifference	0.902 **	0.231	0.443	1.362	0.407
Common Humanity	0.804 *	0.292	0.225	1.384	0.287
Separation	1.033 **	0.230	0.576	1.489	0.468
Mindfulness	0.761 *	0.235	0.295	1.227	0.338
Disengagment	0.772 *	0.237	0.301	1.242	0.340
Total CO	5.000 **	0.934	3.144	6.856	0.558

Note. * *p* < 0.05, ** *p* < 0.001.

**Table 4:** PERMA-Profiler total and subscale paired-sample results.

			95% CI of the Difference		
	Mean Difference	*SE*	LL	UL	Cohen’s *d*
Well-being	12.467	1.669	9.152	15.783	0.779
Negative Emotions	2.239	0.430	1.385	3.093	0.543
Health	1.696	0.358	0.984	2.408	0.493
Loneliness	1.000	0.249	0.506	1.494	0.419
Total	17.402	2.179	13.073	21.730	0.833

Note. All *p*-values < 0.001.

## Data Availability

The datasets presented in this article are not readily available because participants’ approval was not taken for data sharing.
